# Combining Celery Oleoresin, Limonene and Rhamnolipid as New Strategy to Control Endospore-Forming *Bacillus cereus*

**DOI:** 10.3390/foods10020455

**Published:** 2021-02-19

**Authors:** Paula de Camargo Bertuso, Débora M. Drappé Mayer, Marcia Nitschke

**Affiliations:** 1 Interunits Graduate Program in Bioengineering (EESC/FMRP/IQSC), University of São Paulo, Trabalhador São-carlense Av., 400, São Carlos, SP 13566-590, Brazil; paulabertuso@usp.br (P.d.C.B.); debora_mayer@hotmail.com (D.M.D.M.); 2 São Carlos Institute of Chemistry (IQSC), University of São Paulo, Trabalhador São-carlense Av., 400, P.O. Box 780, São Carlos, SP 13560-970, Brazil

**Keywords:** *Bacillus cereus*, rhamnolipid, *Apium graveolens*, oleoresin, limonene

## Abstract

Foodborne diseases (FBD) are a great problem worldwide, leading millions of people to seek medical help and to significant economic losses for industry. Among the agents implicated in FDB is *Bacillus cereus*, a Gram-positive, toxigenic and endospore-forming bacterium. In this study, rhamnolipid (RL) biosurfactant, celery oleoresin (OR) and limonene (LN) were evaluated as bio-based alternatives for controlling the growth of vegetative cells and endospores of *B. cereus*. To address their antimicrobial activity, the compounds were tested separately and in combination. Results demonstrate that, when combined with RL, both OR and LN have lower minimal inhibitory concentration (MIC) values and increased endospore inhibition potential. A percentage of endospore inhibition from 73% to 98%, corresponding to a 2.8–3.6 log reduction in spore outgrowth, was observed. RL inhibited *B. cereus* growth and endospore germination and potentially enhanced the antimicrobial efficacy of the natural hydrophobic compounds tested.

## 1. Introduction

Foodborne diseases (FBD) resulting from the ingestion of foodstuffs contaminated with microorganisms or chemicals represent a growing public health problem worldwide [1]. It is estimated that 600 million people fall sick and 420 thousand die every year after ingestion of contaminated food [2]. Between 2007 and 2017, 96% of FBD outbreaks registered in Brazil were caused by bacteria, including *Bacillus cereus* [3]. In 2016, a food poisoning outbreak was reported in New York from a Chinese fast food chain and 33 *B. cereus* isolates were found on bean samples [4]. In outbreaks registered in France from 2007 to 2014, as little as 400 CFU/g *B. cereus* was found in the incriminated foods and reported to be enough to cause symptoms [5]. Those studies help illustrate how difficult it is to eliminate the bacterium in industrial processing plants and show its capacity to cause disease in humans even when present at low numbers. Considering the importance of controlling such pathogens to public health, food manufactures are continuously searching for innovative methods to guarantee the safety of their products.

Consumers’ preference for natural additives rather than artificial ones [6] stimulates the industry to find new bio-based and green preservatives. The use of natural biocontrol agents derived from microbes or plants such as bacteriocins, endolysins and essential oils (EOs) is gaining increasing interest, especially due to the development of microbial tolerance to disinfectants [7].

EOs are complex, volatile and hydrophobic compounds formed by plants as secondary metabolites [8,9]. Their antimicrobial activity against food pathogens has been extensively reported [10,11,12,13]. Oleoresins (OR), in contrast, are viscous mixtures of essential oils and resins that are extracted from spices through organic solvents [14,15]. ORs can be found in liquid form when adding solvents such as propylene glycol, which also facilitates their use in food products [14]. ORs contain both volatile and non-volatile components and present a high shelf life since they are practically absent of water, thus reducing oxidative degradation, flavor loss and microbial contamination [16]. D-limonene (LN) is the major constituent of several EOs, especially from citrus species, and its presence has been associated with the antimicrobial activity of such compounds [11,12].

Although plant-derived oils and oleoresins have shown antimicrobial effects, their use is limited, due to the higher concentrations needed to be effective, their low water solubility and their strong sensorial impact [17]. An alternative to this problem is the use of emulsifiers, such as surfactants, that permit improving water solubility, reducing the amount of the oil needed and its undesirable sensorial effects.

Microbial-derived surfactants can replace synthetic ones with some advantages since they show similar surfactant and emulsifier characters, are eco-friendly and bio-based and are good candidates in the development of innovative “green” food products [18]. Rhamnolipids (RL) are glycolipid biosurfactants, produced primarily by *Pseudomonas* sp., that have shown potential as antimicrobial, anti-adhesive and anti-biofilm agents against food pathogens [19,20,21,22].

Within this context, this work investigates the antimicrobial potential of celery (*Apium graveolens*) OR, limonene and their combination with rhamnolipids against planktonic cells and endospores of *B. cereus*.

## 2. Materials and Methods

### 2.1. Oils, Oleoresins and Biosurfactant

Oleoresin (8%) extracted from *Apium graveolens* seeds was kindly donated by Beraca Sabará S.A (Santa Bárbara d’Oeste, Brazil). Limonene (97% purity—Cutrale, Araraquara, Brazil) was kindly donated by Prof. André L. M. Porto. Rhamnolipid biosurfactant (90% purity) was acquired from AGAE Technologies (Corvallis, OR, USA).

### 2.2. Mixture Stock Solutions

OR or LN was mixed with propylene glycol (1:1 or 1:0.5 for endospores germination experiment) before being added to the culture broth containing a final concentration of 0.02% of Tween 80 or 500 µg/mL of RL. The final concentration for OR and LN in stock solutions was 80,000 µg/mL and such values were based on the maximum amount of propylene glycol found to not affect bacterial growth, which was previously defined as 12.5%.

The mixtures were homogenized by vortexing and filtered (0.45 µm). RL was diluted in culture broth and further sterilized by filtration (0.22 μm). The final concentration of RL stock solution was 5000 µg/mL.

### 2.3. Microorganism

The *Bacillus cereus* ATCC 33,018 strain was stored at −20 °C on TSB (Tryptic Soy Broth—Himedia) supplemented with 6 g/L of yeast extract (TSYEB) and 20% (*v*/*v*) glycerol.

### 2.4. Identification of Celery Oleoresin Components by GC-MS

Fatty acid methyl esters (FAME) of celery OR (10 mg) were prepared under stirring using concentrated sulfuric acid (1 drop) and methanol (1 mL) at 60 °C for 30 min. After cooling, 2 mL of NaCl and 1 mL of n-hexane were added, and the solution was stirred and left undisturbed for phase separation. The upper phase was separated, and chromatography analysis was performed using a Shimadzu QP2010 (Shimadzu Corporation, Kyoto, Japan) system comprising an AOC-20 auto-sampler and gas chromatograph interfaced with a mass spectrometer (GC-MS QP2010 Plus) with a J&W Scientific DB-5MS (Folsom, CA, USA) (5% phenylmethylpolysiloxane) fused silica capillary column (30 cm × 0.25 mm i.d., 0.25 µm film thickness). The operation was carried out in electron impact mode at 70 eV. Helium (99.999%) was used as carrier gas at a constant flow of 0.99 mL/min, and an injection volume of 1 μL was employed (split ratio of 1:10). The injector temperature was 250 °C, and the ion source temperature was 250 °C. The oven temperature was programmed from 50 (isothermal for 1 min), with an increase of 5 °C/min., to 300 °C, ending with a 10-min isothermal period at 300 °C. Mass spectra were taken at 70 eV with a scan interval of 0.3 s and fragments of 40 to 500 Da [23]. The identification of the methyl esters was performed by computerized matching of the acquired mass spectra with those stored in mass spectral libraries of the equipment data system. For terpene analysis, a mixture of hydrocarbons (C_9_H_20_–C_18_H_38_) was injected under the conditions described above and identification of constituents was also performed by computerized matching and by comparison of the spectra obtained with those of the databank and considering the relative retention index (RRI) [24].

### 2.5. Determination of Minimal Inhibitory Concentration (MIC) and Minimal Bactericidal Concentration (MBC)

Stock cultures of *B. cereus* were transferred to TSEYA (Tryptic Soy Agar supplemented with 6 g/L of yeast extract) and incubated for 24 h at 37 °C. Cells were transferred to 5 mL of TSEYB and incubated for an additional 24 h at 37 °C. An aliquot of 1 mL of cell suspension was transferred to 4 mL of fresh TSEYB and incubated for 3 h at 37 °C. Antimicrobial activity was performed on 96-well microplates using the micro-broth dilution technique based on Clinical and Laboratory Standards Institute [25] guidelines. Microplate wells were filled with 100 μL of TSEYB followed by addition of 100 μL of the tested mixture on the first column and a 2-fold serial dilution. After serial dilutions of the antimicrobials, 20 µL of the standardized bacterial inoculum (10^7^ CFU/mL) was added to each well and the microplates were incubated at 37 °C for 24 h. After visual inspection, 10 µL from the wells where no bacterial growth was observed was spotted on agar plates and incubated at 37 °C for 24 and 48 h. MBC was defined as the lowest MIC concentration where no viable growth was detected. Subsequently, 20 µL of 0.1% tetrazolium bromide (MTT-Sigma Aldrich, St. Louis, MO, USA) solution was added to the wells for 1 h to confirm the presence or absence of growth. The MIC was defined as the lowest concentration of antimicrobial mixture that showed no change in the MTT original color.

### 2.6. Time–Kill Assay

The bacterial growth in the presence of antimicrobials was evaluated using a time dependence assay as described by Verma, 2007 [26]. The tests were conducted in glass tubes filled with 5 mL of culture broth containing one of the antimicrobial mixtures (RL, OR + RL or LN + RL) at MIC and 16x MIC concentration. Inoculum was prepared as described above (2.5) and 1 mL of standardized bacterial suspension was added to the tubes following incubation at 37 °C. At different time intervals, the number of viable cells was determined by the drop method [27]. Control tubes with no antimicrobial addition were also assessed.

### 2.7. Endospore Germination Inhibition

Stock cultures of *B. cereus* were transferred to TSEYA and incubated for 24 h at 37 °C. After that, cells were transferred to a modified nutrient agar plate to favor sporulation (nutrient agar supplemented with 0.06 g/L MgSO_4_ and 0.35 g/L KH_2_PO_4_) and incubated for 10 days at 37 °C [28].

Resulting cultures were suspended in sterilized distilled water, and the optical density was adjusted to 0.5 at 610 nm. This OD corresponds to 3 × 10^7^ cells/mL. Cell suspension was then heated to 75 °C for 20 min to assure only the endospores were present [28]. To confirm sporulation, malachite green staining was performed (2.8). An amount of 1 mL of endospore suspension was then added to 5 mL of TSEYB containing specific concentrations (16× MIC and 32× MIC) of RL, OR + RL or LN + RL. Samples were further incubated at 37 °C without agitation. At specific time points (0, 2, 6, 10 and 24 h), a 0.1 mL aliquot from each treatment was taken and added to 0.9 mL of saline solution (NaCl 0.86%) to perform a 10-fold serial dilution. Viable cells were counted by using the drop method [27]. Non-treated cells at time 0 were used as control for germination inhibition experiments. Log reduction was calculated for each specific time comparatively to the control.

The percentage of endospore germination inhibition and the log reduction were calculated using the following equations [28]:

(1) $$\%\;\mathrm{endospore}\;\mathrm{germination}\;\mathrm{inhibition}\;=\;\frac{\left(CFUcontrol-CFUtreated\right)}{CFUcontrol}\times 100,$$

(2) $$\mathrm{Log}\;\mathrm{reduction}\;=\;\log_{10}\left(\frac{CFUcontrol}{CFUtreated}\right)$$

### 2.8. Endospore Staining

A 2 mL sample of culture broth was centrifuged at 10,000 rpm for 10 min. The supernatant was discarded, and cells were washed with 2 mL distilled water and centrifuged again. Supernatant was discarded and cell pellet was resuspended in 50 μL distilled water. Cellular suspension was then transferred to a glass slide, heat fixed and covered with malachite green solution (5%). Slides were passed through a flame for 5 min, paying attention to not boil the dye. The slide was then washed and stained with a safranin solution (2.5%) for 30 s [29,30]. After being air dried, the slides were observed by bright-field microscopy using immersion lens.

### 2.9. Statistics

MIC and MBC values were expressed as the mode of at least three independent replicates. All other data are represented as the mean ± SD of three independent replicates. Analysis of variance (ANOVA) and the Tukey test were performed using OriginPro, version 8.5 (OriginLab Corporation, Northampton, MA, USA).

## 3. Results and Discussion

A preliminary screening using food-derived essential oils and oleoresins showed that *Apium graveolens* OR was efficient in controlling the growth of food pathogens such as *Listeria monocytogenes* and *Bacillus cereus* (data not shown). Based on such previous observations, we developed this new study to investigate, in detail, the antimicrobial activity demonstrated by the celery OR. Considering that one of the main difficulties in the application of EO and OR is their low water solubility, we also evaluated their combination with rhamnolipids since the amphiphilic nature of the biosurfactant may improve solubility and favor the delivery of hydrophobic compounds to cell targets. In addition, rhamnolipids also demonstrate antimicrobial activity against several Gram-positive food pathogens [31]; thus, we hypothesize that their combination with OR and/or EO can improve the antimicrobial potential of the natural compounds. *Bacillus cereus* was selected as our model study bacterium because of its importance as a food pathogen, along with its endospore-forming ability.

### 3.1. Analysis of Celery Oleoresin

To identify the main components present in the celery OR, a GC-MS analysis was performed and the results are shown in Table 1. Terpenes (limonene), sesquiterpenes (β-selinene), flavor (aromatic) compounds (3-buthylphthalide, sedanenolide) and fatty acid esters were found as the major compounds. Although no reports using oleoresin are available for comparison, most studies in the literature identified terpenes and sesquiterpenes as the main active components of celery seed EO [32,33]. Therefore, we also investigated the antimicrobial activity of limonene (LN) since it is considered the major component of celery EO responsible for the antimicrobial activity against several pathogens [33,34].

### 3.2. Minimal Inhibitory Concentration (MIC) and Minimal Bactericidal Concentration (MBC)

Vegetative B. cereus cells were treated with solutions containing RL, OR and LN alone or in combination to determine the minimal concentrations necessary to inhibit growth and to kill the bacterial cells after 24 and 48 h of exposure (Table 2).

When considered alone, RL was able to inhibit cell growth with concentrations as low as 9.8 μg/Ml, and cell death was obtained with 156.25 μg/mL. OR showed the highest MIC value of tested compounds. For both oils, it was not possible to determine the MBC based on the concentrations tested. When combined with RL, on the other hand, both OR and LN showed a reduction in the MIC and present MBC. The mixture containing OR + RL was able to inhibit cell growth with 2500 + 15.63 μg/mL and kill cells with 20,000 + 125 μg/mL (24 h), while the LN + RL mixture had an MIC of 1250 + 7.81 μg/mL and an MBC of 20,000 + 125 μg/mL. These results suggest that, when combined with RL, the antimicrobial effect of OR and LN is enhanced. By contrast, it is also possible to assume that OR may have an inhibitory effect on RL, since Table 2 shows that more RL is needed to reach the MIC when combined with OR.

### 3.3. Time–Kill Assays

A new set of experiments was conducted using the previously determined MIC and MBC values, in order to study the bacterial behavior in the presence of the antimicrobials during the time-defined intervals. Since no MBC was observed even for OR and LN alone, they were not considered for this assay.

**Figure 1:**
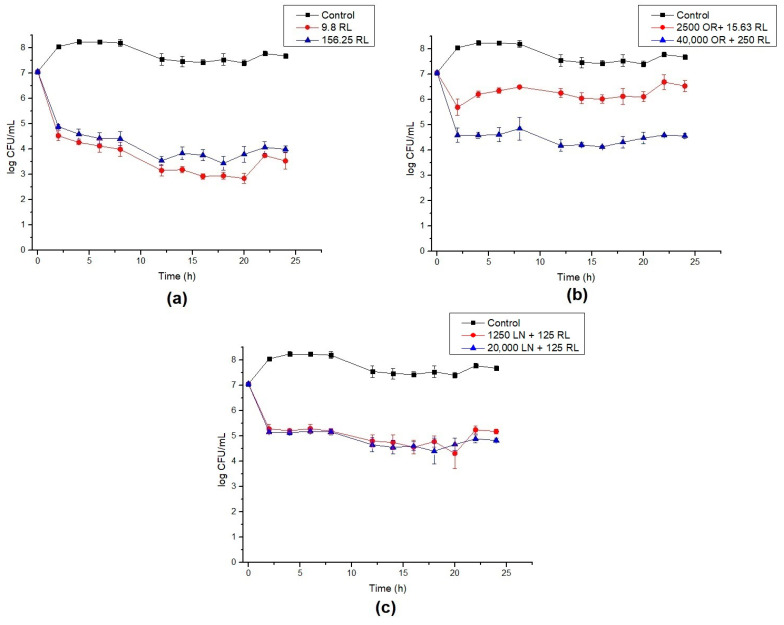
Time–kill curves for *B. cereus* with (**a**) rhamnolipid (RL), (**b**) celery oleoresin (OR) + RL and (**c**) limonene (LN) + RL. Lines show the log CFU/mL for control (black), minimum inhibitory concentration (MIC) (red) and 16× MIC (blue). Error bars show the standard deviation of at least three independent replicates.

The growth of *B. cereus* cells in the presence of RL, OR + RL and LN + RL revealed that the values initially designated as MBC are not capable of eradicating the cell population (Figure 1), although they were 16 times higher than the MIC concentration. Rhamnolipid treatment reduced the viable cell population by around 4 log and the population was maintained at this level after 24 h (Figure 1a). The combination of RL with LN and OR improved their antimicrobial efficacy, corroborating the data shown in Table 2. For LN + RL, there is no apparent difference between the MIC and the 16x MIC values (Figure 1c). On the contrary, for OR + RL, it is possible to observe that the increase in the mixture concentration caused a decrease in cell survival (Figure 1b). This fact can be explained by the increasing concentration of active compounds present in OR, such as terpenes and sesquiterpenes (Table 1).

**Figure 2:**
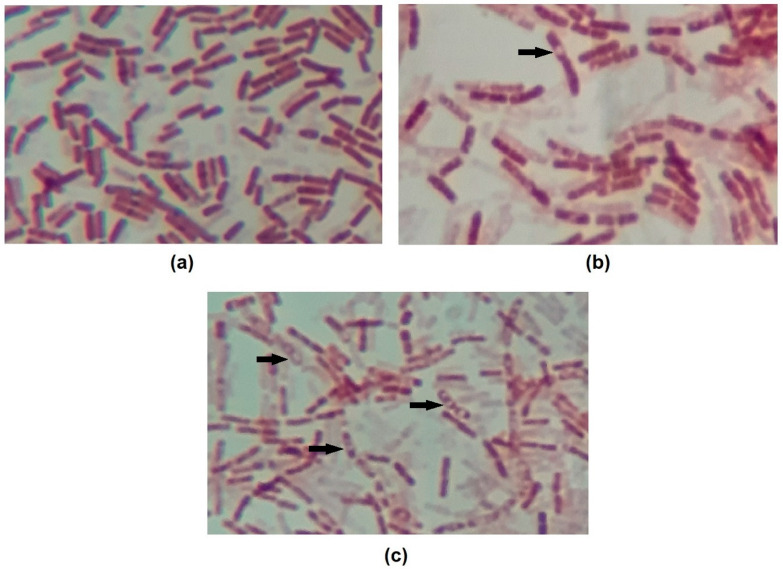
Images of endospore staining of (**a**) control, (**b**) OR + RL MIC 14h and (**c**) OR + RL MIC 24h treatments. Endospores are visible on images (**b**) and (**c**) as an uncolored region inside the cells. Some endospores are indicated by arrows. Magnification 1000×.

It is also worth noting that all curves show roughly a 2 log drop in CFU/mL after only 2 h of contact. We speculate that this behavior is caused by the endospore-forming ability of *B. cereus* under adverse situations. The initial drop in cell counts means that some cells died within this period of time, while the remaining cells switched on into endospores. To confirm this hypothesis, test samples were stained, and microscopy revealed the presence of endospores (Figure 2).

The inconsistency in MBC values described in Table 2 can be explained by the count method utilized. We observed that during serial dilutions to perform the viable count, the first undiluted sample did not show growth after incubation; however, in the subsequent decimal dilutions of the same sample, several colonies were observed (Supplementary Materials Figure S1). This fact is caused by the antimicrobial dilution factor; thus, in the first sample (without dilution), the drug concentration was able to avoid cell growth, while after dilution, the remaining cells (or endospores) could grow. As the MBC was performed after 24 and 48 h of incubation by plating the original sample in a fresh plate without dilution, the remaining antimicrobial concentration was sufficient to inhibit the growth or, possibly, endospore germination. In addition, as shown in Table 2, the MBC of the OR + RL mixture was increased after 48 h, corroborating the statements described above.

Antimicrobial activity against *Bacillus cereus* was previously reported for RL [31,35], limonene [36,37] and celery EO [35]; however, there are no reports regarding the use of celery oleoresin and/or EO components associated with RL. Similar studies using the particular compounds showed that celery seed EO (100% *v*/*v*) presented an inhibition zone of 33 mm against *B. cereus* cells [32]. In another study, 250 μg/mL of limonene was able to inhibit *B. cereus* growth; however, no MBC was observed [37]. In addition, an inhibition zone of 6.3–6.7 mm was reported when 420 μg of limonene was tested against two strains of *B. cereus* [36]. Rhamnolipids showed an MIC of 19.5 μg/mL [31] and an inhibition zone ranging from 13 to 22 mm against *B. cereus* strains [35]. These examples point out that differences regarding the strains’ sensitivity, methodology, purity of the compounds and their formulations can influence the antimicrobial activity; thus, it is difficult to compare our results with the ones available in the literature.

Considering that the mechanism involved in the antimicrobial activity of both RL and LN/EO is related to disturbance in the permeability/integrity of the bacterial cell membrane [34,38,39,40], we can suggest that the combination of a biosurfactant and active hydrophobic compounds may in fact increase their solubility, favoring interaction with the cells.

As observed, the presence of RL and its combination with OR and LN can inhibit the growth of vegetative cells but also induces *B. cereus* sporulation (Figure 2). It was also demonstrated that under the presence of the antimicrobials, the population was maintained relatively constant, suggesting they might also inhibit endospore germination. To test this hypothesis, further germination inhibition assays were conducted.

### 3.4. Endospore Inhibition

**Figure 3:**
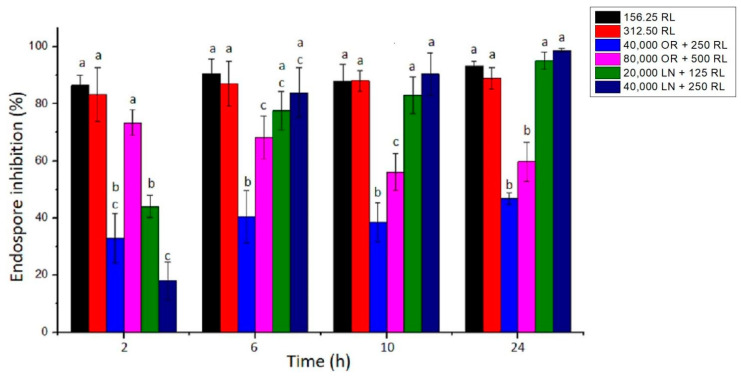
Percentage of *B. cereus* endospore germination inhibition of RL, OR + RL and LN + RL treatments. The concentrations used for this experiment were 16× and 32× MIC. Error bars represent the standard deviation of at least three independent replicates. For each time of exposure, treatment with the same letters did not differ significantly (*p* < 0.05).

**Figure 4:**
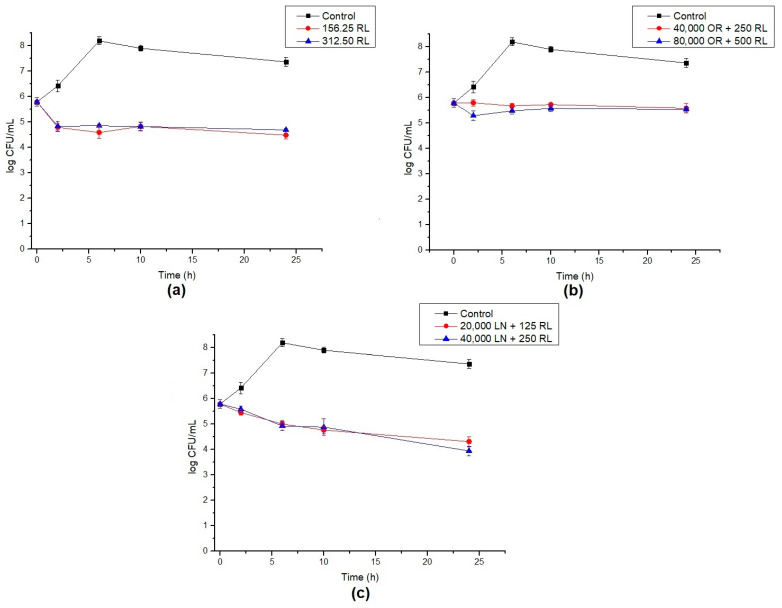
Log reduction in endospore germination after treatment with (**a**) RL, (**b**) OR + RL and (**c**) LN + RL. Lines show the log CFU/mL for control (black), 16× MIC (red) and 32× MIC (blue). Error bars show the standard deviation of at least three independent replicates.

The endospores of *B. cereus* were treated with RL and its respective mixtures with celery OR and LN. The percentage of germination inhibition (CFU) and log reduction in outgrowth were calculated after different times of exposure. RL showed a reduction around 93% in germination after 24 h. Additionally, the increase in RL concentration and time of exposure did not significantly increase the inhibition of endospore germination (Figure 3). A maximum of a 3.6 log reduction in endospore outgrowth was also observed after 6 h compared to the control at the same time (Figure 4a). When RL was mixed with OR, the maximum inhibition percentage (73%) and log reduction (2.8) were obtained for the highest concentration tested. As also shown in Figure 1b, the increase in concentration improved the activity of the OR + RL mixture, probably due to the increase in the active compounds present in OR (Table 1). As observed in vegetative cells, OR also seems to inhibit the effect of RL on endospores, since when both antimicrobials are combined, lower endospore germination inhibition values are displayed compared to RL alone (Figure 1).

The combination of LN + RL demonstrated the highest endospore inhibition values after 24 h of exposure, achieving a maximum of 98% inhibition. An important decrease in cell counts over time was also observed, reaching a 3.3 log reduction after 24 h, as shown in Figure 4c. As previously discussed, one of the main components of OR is LN (around 9.5%) and it may be responsible (at least in part) for OR’s antimicrobial effect. This could explain why, when LN is in its purified form, it shows higher antimicrobial potential compared with OR samples.

Regardless of RL and EO, antimicrobial action against vegetative *B. cereus* cells is well documented, though reports on their activity against endospores are scarce in the literature. Huang et al., 2007 [41], evaluated the potential of a lipopeptide biosurfactant produced by *B. subtilis* to inactivate *B. cereus* endospores. The lipopeptide displayed an MIC of 156.25 μg/mL, and the authors proposed the combination with heat to reduce endospore germination. A 2 log reduction was attained after treatment of *B. cereus* endospores for 7.6 h at 29.6 °C with 3.46 mg/mL of the lipopeptide. A spore coat disruption was also observed, and the authors proposed that the surfactant nature of the compound could favor the binding to lipoproteins and internalization of the biosurfactant, resulting in coat damage.

The increasing in lipophilic character of organic acids and alcohols enhanced the inhibition of *B. cereus* spore germination. The accumulation of such compounds in the inner membrane was correlated with the length of their carbon chains and consequent endospore germination inhibition [42].

Further reports have demonstrated that the amphiphilic nature of surfactants, more precisely, their hydrophilic–lipophilic balance (HBL), can be correlated with endospore inactivation potential. The hydrophobic compounds citral, *p*-cymene and bornyl acetate were more effective at inhibiting *B. subtilis* endospores than hydrophilic compounds such 2,3 dihydrobenzofuran and β-pinene [43]. The average HBL value of the compounds causing a significant 1–2 log reduction in endospores was 9.3, which is similar to several chemical surfactants. According to the authors, the structural proteins of the spore coat can be changed due to binding of their polar and apolar groups to hydrophilic and hydrophobic moieties of the surfactants.

Green tea polyphenol samples including a crude extract, epigallacatechin-3-gallate and their respective lipophilic derivatives were reported to prevent endospore germination in different *Bacillus* species. The four types of polyphenols inhibited *B. cereus* spore germination by 94–100% with a log reduction ranging from 1.27 to 3.0. A disruption in the *B. cereus* spore coat, changing in coat morphology and spore agglutination, was observed after the polyphenol treatment [28].

Although the exact mechanisms involved in spore inhibition are unknown, it is a consensus that the lipophilic nature of chemical compounds influences their activity. Since the endospore surface is hydrophobic, molecules with this character might easily interact with several targets present in the inner/outer coats, cortex, membrane and core [44].

The HBL of RL is dependent (among other factors) on the purity and composition of homologous compounds present in the mixture, and several different values have been reported in the literature, ranging from 6.5 to 24 [45,46]. Thus, the amphiphilic character presented by the rhamnolipids may account for the results observed in our work. The combination of RL with the hydrophobic compounds LN and OR can improve their solubility and consequent interaction with endospore structures, as also proposed for vegetative cells. Our results demonstrate that RL potentially inhibits *B. cereus* vegetative cells and endospore germination and also enhances the antimicrobial action of the oil-derived compounds tested. Further studies should be conducted to understand the mechanism involved in the endospore inhibition by the compounds.

To our knowledge, this is the first report on using RL and its mixture with LN and celery OR to inhibit *B. cereus* endospores. Combinations of such compounds with physical methods (heat, radiation, pressure) or the development of micro/nanoemulsions exploring RL and OR/EO components may result in innovative strategies to control this important food pathogen.

## Figures and Tables

**Table 1:** Chemical composition of celery (*Apium graveolens*) oleoresin.

	RT (min) ^1^	Compound	% GC-MS	Exp RRI ^2^	Lit RRI ^3^
1	9.965	limonene	9.48	1039	1024
3	13.415	pentyl ciclohexa-1,3-diene	0.36	1158	1156
4	18.980	1-phenyl-1-pentanone	0.57	1358	1364
5	20.705	β-kariophylene	0.23	1423	1417
6	22.455	β-selinene	3.11	1494	1489
7	22.615	α-selinene	0.77	1501	1498
8	23.190	methyl dodecanoate	0.06	1524	--
11	24.725	kariophylene oxide	0.27	1586	1582
12	26.295	3-butylphthalide	5.67	1655	1647
13	26.415	β-eudesmol	0.86	1661	1649
14	26.730	3Z-butylidenephthalide	0.20	1675	1671
16	27.765	sedanenolide	7.41	1722	1719
17	27.915	neocnidilide	1.75	1728	1722
18	28.040	Z-ligustilide	0.34	1734	1734
19	29.980	methyl pentadecanoate	0.27	1815	--
23	31.435	methyl 7,10,13-hexadecatrienoate	0.28	1859	--
24	31.535	methyl 7-hexadecenoate	0.37	1862	--
25	31.600	methyl 9-hexadecenoate	0.87	1865	--
27	32.0.45	methyl hexadecanoate	8.23	1878	--
28	32.745	hexadecanoic acid	0.08	1900	--
31	33.985	methyl heptadecanoate	0.26	1947	--
35	35.280	methyl 9,12-octadecadienoate	11.68	1996	--
36	35.465	methyl 9-octadecenoate	30.50	2003	--
37	35.875	methyl octadecanoate	3.58	2019	--
38	36.115	9-octadecenoic acid	1.03	2028	--
40	38.880	methyl 11-eicosenoate	0.23	2134	--
42	39.375	methyl eicosanoate	0.66	2152	--
44	41.030	methyl henicosanoate	0.08	2216	--
47	42.620	methyl docosanoate	0.56	2276	--
48	44.155	methyl tricosanoate	0.18	2334	--
52	45.630	methyl tetracosanoate	0.58	2391	--
53	46.785	trans-squalene	0.14	2435	--
54	47.065	methyl pentacosanoate	0.08	2445	--
57	48.440	methyl hexadocosanoate	0.40	2498	--
60	51.070	methyl octacosanoate	0.49	2598	--
61	52.705	β-stigmasterol	0.51	2660	--
63	54.045	methyl triacontanoate	0.16	2711	--
Identified			92.30		
Non-identified			7.70		
Fatty esters			60.63		
Total			100.00		

^1^ Retention time in minutes. ^2^ Experimental relative retention index. ^3^ Literature relative retention index.

**Table 2:** Antimicrobial activity of the tested compounds against *B. cereus* vegetative cells.

Compound	MIC (μg/mL)	MBC 24h (μg/mL)	MBC 48h (μg/mL)
RL	9.8	156.25	156.25
OR	40,000	>40,000	>40,000
OR + RL	2500 OR + 15.63 RL	20,000 OR + 125 RL	>40,000 OR + 250 RL
LN	2500	>40,000	>40,000
LN+RL	1250 LN + 7.81 RL	20,000 LN + 125 RL	20,000 LN + 125 RL

## Data Availability

The data presented in this study are available on request from the corresponding author.

## Supplementary Materials

The following are available online at https://www.mdpi.com/2304-8158/10/2/455/s1, Figure S1: Images of the Petri dishes used for viable cell counting. On the undiluted sample, growth after incubation was not observed; however, as the dilution of the sample occurred, several colonies were observed. (**a**) LN + RL; (**b**) RL. Numbers on plates represent how many times the samples were 10-fold diluted.
