# Correction: Bertuso et al. Combining Celery Oleoresin, Limonene, and Rhamnolipid as a New Strategy to Control Endospore-Forming *Bacillus cereus. Foods* 2021, *10*, 455

**DOI:** 10.3390/foods12030645

**Published:** 2023-02-02

**Authors:** Paula de Camargo Bertuso, Débora M. Drappé Mayer, Marcia Nitschke

**Affiliations:** 1 Interunits Graduate Program in Bioengineering (EESC/FMRP/IQSC), University of São Paulo, Trabalhador São-Carlense Av., 400, São Carlos, SP 13566-590, Brazil; 2 São Carlos Institute of Chemistry (IQSC), University of São Paulo, Trabalhador São-Carlense Av., 400, P.O. Box 780, São Carlos, SP 13560-970, Brazil

## Text Correction

There was an error in the original publication. The real concentration of RL is actually 10 times higher than the one previously reported.

Corrections were made to “2.2. Mixture Stock Solutions” and “3.2. Minimal Inhibitory Concentration (MIC) and Minimal Bactericidal Concentration (MBC)”.

### 2.2. Mixture Stock Solutions

OR or LN was mixed with propylene glycol (1:1 or 1:0.5 for the endospores germination experiment) before being added to the culture broth containing a final concentration of 0.02% of Tween 80 or 5000 µg/mL of RL. The final concentration of OR and LN in the stock solutions was 80,000 µg/mL, and such values were based on the maximum amount of propylene glycol determined not to affect the bacterial growth, which was previously defined as 12.5%.

The mixtures were homogenized by vortexing and filtered (0.45 µm). RL was diluted in culture broth and further sterilized by filtration (0.22 μm). The final concentration of RL stock solution was 50,000 µg/mL.

### 3.2. Minimal Inhibitory Concentration (MIC) and Minimal Bactericidal Concentration (MBC)

Vegetative *B. cereus* cells were treated with solutions containing RL, OR, and LN, alone or in combination, to determine the minimal concentrations necessary to inhibit growth and to kill the bacterial cells after 24 and 48 h of exposure (Table 2).

When considered alone, RL was able to inhibit cell growth with concentrations as low as 98 μg/mL, and cell death was obtained with 1562.5 μg/mL. OR showed the highest MIC value of the tested compounds. It was not possible to determine the MBC of either oil based on the concentrations tested. When combined with RL, on the other hand, both OR and LN showed a reduction in the MIC and exhibited MBC. The mixture containing OR + RL was able to inhibit cell growth at 2500 + 156.3 μg/mL and to kill cells at 20,000 + 1250 μg/mL (24 h), while the LN + RL mixture had an MIC of 1250 + 78.1 μg/mL and an MBC of 20,000 + 1250 μg/mL. These results suggest that, when combined with RL, the antimicrobial effect of OR and LN is enhanced. By contrast, it is also possible to assume that OR may have an inhibitory effect on RL, since Table 2 shows that more RL is needed to reach the MIC when combined with OR.

## Error in Figures/Table

In the original publication [1], there was a mistake in Table 2 and Figures 1, 3 and 4, as published. In the aforementioned table and figures, the concentration of RL was 10 times lower than the real concentration. The corrected Table 2 and Figure 1, Figure 3 and Figure 4 appear below. The authors state that the scientific conclusions are unaffected. This correction was approved by the Academic Editor. The original publication has also been updated.

## Figures and Tables

**Figure 1:**
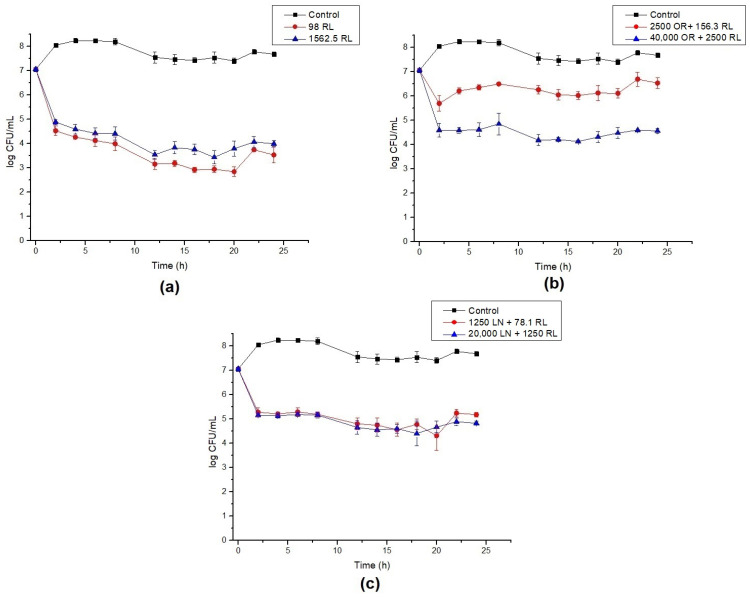
Time–kill curves for *B. cereus* with (**a**) rhamnolipid (RL), (**b**) celery oleoresin (OR) + RL, and (**c**) limonene (LN) + RL. Lines show the log CFU/mL for the control (black), minimum inhibitory concentration (MIC) (red), and 16× MIC (blue). Error bars show the standard deviation of at least three independent replicates.

**Figure 3:**
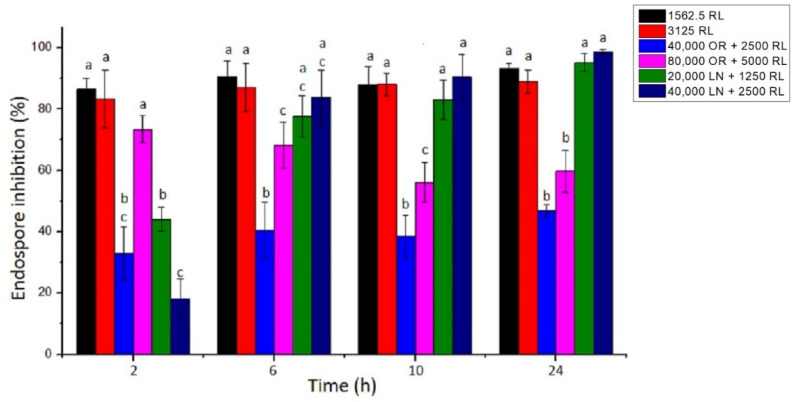
Percentage of *B. cereus* endospore germination inhibition of RL, OR + RL, and LN + RL treatments. The concentrations used for this experiment were 16× and 32× MIC. Error bars represent the standard deviation of at least three independent replicates. For each time of exposure, treatments with the same letters did not differ significantly (*p* < 0.05).

**Figure 4:**
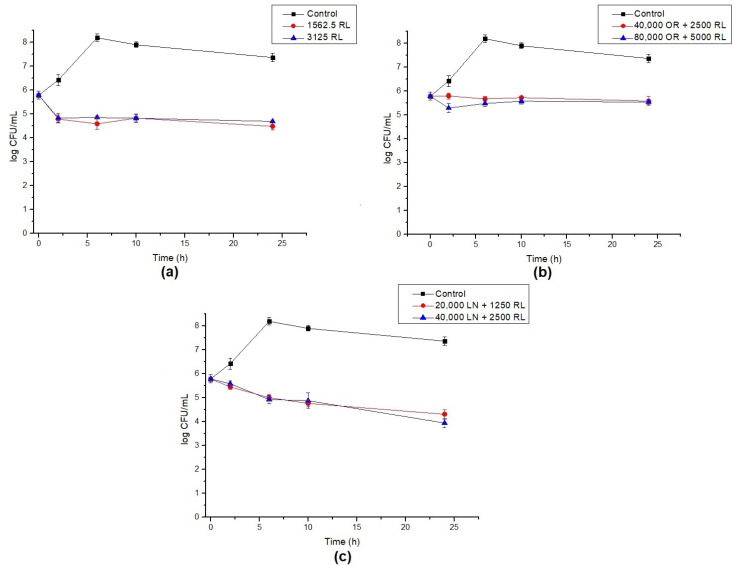
Log reduction in endospore germination after treatment with (**a**) RL, (**b**) OR + RL, and (**c**) LN + RL. Lines show the log CFU/mL for the control (black), 16× MIC (red), and 32× MIC (blue). Error bars show the standard deviation of at least three independent replicates.

**Table 2:** Antimicrobial activity of the tested compounds against *B. cereus* vegetative cells.

Compound	MIC (μg/mL)	MBC 24 h (μg/mL)	MBC 48 h (μg/mL)
RL	98	1562.5	1562.5
OR	40,000	>40,000	>40,000
OR + RL	2500 OR + 156.3 RL	20,000 OR + 1250 RL	>40,000 OR + 2500 RL
LN	2500	>40,000	>40,000
LN + RL	1250 LN + 78.1 RL	20,000 LN + 1250 RL	20,000 LN + 1250 RL

**Table 2:** Antimicrobial activity of the tested compounds against *B. cereus* vegetative cells.

Compound	MIC (μg/mL)	MBC 24 h (μg/mL)	MBC 48 h (μg/mL)
RL	98	1562.5	1562.5
OR	40,000	>40,000	>40,000
OR + RL	2500 OR + 156.3 RL	20,000 OR + 1250 RL	>40,000 OR + 2500 RL
LN	2500	>40,000	>40,000
LN + RL	1250 LN + 78.1 RL	20,000 LN + 1250 RL	20,000 LN + 1250 RL
