# Supplementary figures and images for: Combining Celery Oleoresin, Limonene and Rhamnolipid as New Strategy to Control Endospore-Forming Bacillus cereus
Source: Foods. 2021 Feb 19;10(2):455. doi: 10.3390/foods10020455 (PMC7922389; doi:10.3390/foods10020455)

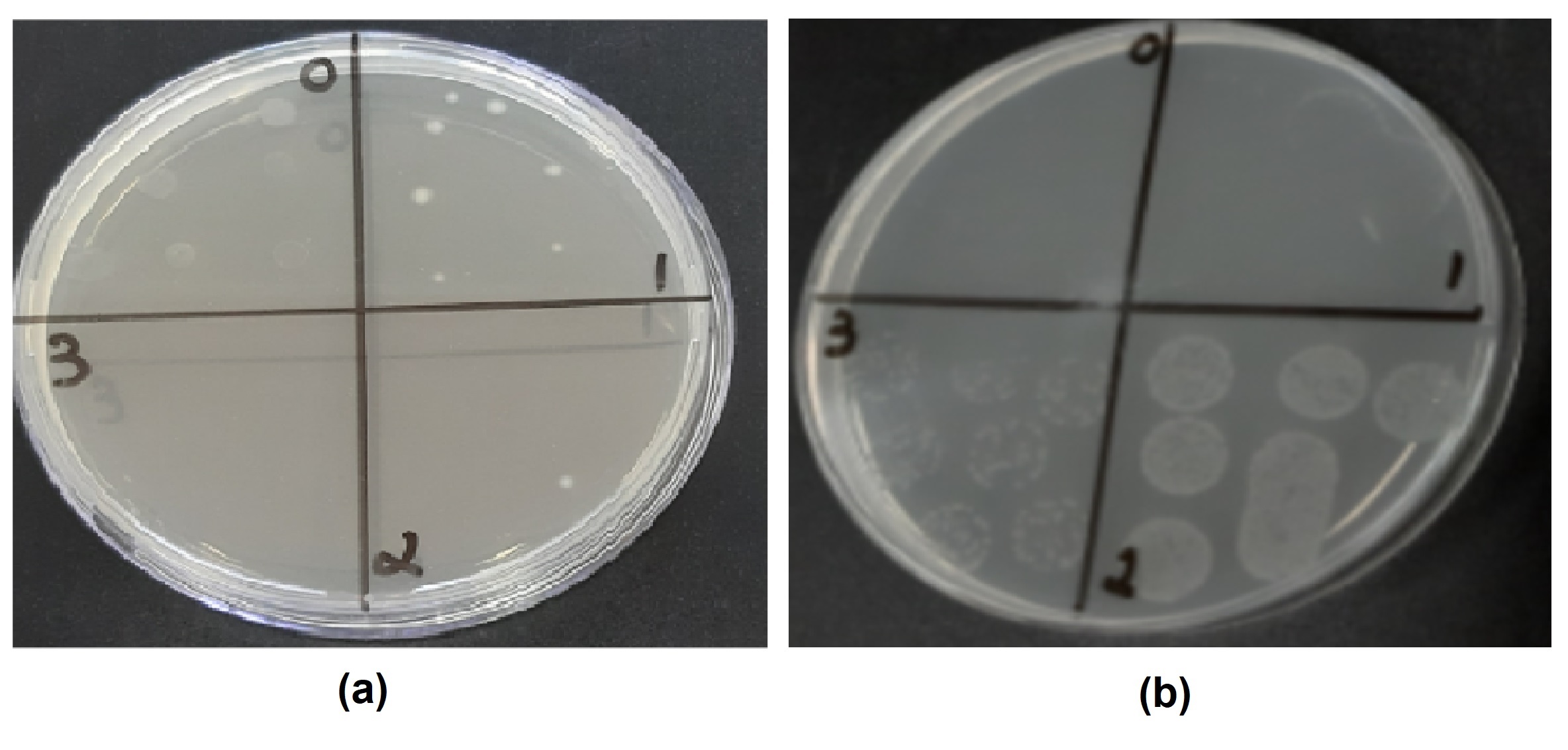

Supplement: Supplementary file 1 [file foods-10-00455-s001.zip › foods-1049974-supplementary.jpg]
